# Weight loss after Roux-En-Y gastric bypass surgery reveals skeletal muscle DNA methylation changes

**DOI:** 10.1186/s13148-021-01086-6

**Published:** 2021-05-01

**Authors:** Luis A. Garcia, Samantha E. Day, Richard L. Coletta, Baltazar Campos, Tonya R. Benjamin, Eleanna De Filippis, James A. Madura, Lawrence J. Mandarino, Lori R. Roust, Dawn K. Coletta

**Affiliations:** 1 Department of Medicine, Division of Endocrinology, The University of Arizona College of Medicine, 1501 N. Campbell Ave, PO Box 245035, Tucson, AZ 85724-5035 USA; 2 Phoenix Epidemiology and Clinical Research Branch, National Institute of Diabetes and Digestive and Kidney Diseases, National Institutes of Health, Phoenix, AZ USA; 3 Department of Endocrinology, Metabolism and Diabetes, Mayo Clinic Arizona, Scottsdale, AZ USA; 4 Mayo Clinic Hospital, Phoenix, AZ USA; 5 Department of Physiology, The University of Arizona College of Medicine, Tucson, AZ USA

**Keywords:** DNA methylation, Next generation sequencing, Skeletal muscle, Obesity, Bariatric surgery

## Abstract

**Background:**

The mechanisms of weight loss and metabolic improvements following bariatric surgery in skeletal muscle are not well known; however, epigenetic modifications are likely to contribute. The aim of our study was to investigate skeletal muscle DNA methylation after weight loss induced by Roux-en-Y gastric bypass (RYGB) surgery. Muscle biopsies were obtained basally from seven insulin-resistant obese (BMI > 40 kg/m^2^) female subjects (45.1 ± 3.6 years) pre- and 3-month post-surgery with euglycemic hyperinsulinemic clamps to assess insulin sensitivity. Four lean (BMI < 25 kg/m^2^) females (38.5 ± 5.8 years) served as controls. We performed reduced representation bisulfite sequencing next generation methylation on DNA isolated from the *vastus lateralis* muscle biopsies.

**Results:**

Global methylation was significantly higher in the pre- (32.97 ± 0.02%) and post-surgery (33.31 ± 0.02%) compared to the lean (30.46 ± 0.02%), *P* < 0.05. MethylSig analysis identified 117 differentially methylated cytosines (DMCs) that were significantly altered in the post- *versus* pre-surgery (Benjamini–Hochberg *q* < 0.05). In addition, 2978 DMCs were significantly altered in the pre-surgery obese *versus* the lean controls (Benjamini–Hochberg *q* < 0.05). For the post-surgery obese *versus* the lean controls, 2885 DMCs were altered (Benjamini–Hochberg *q* < 0.05). Seven post-surgery obese DMCs were normalized to levels similar to those observed in lean controls. Of these, 5 were within intergenic regions (chr11.68,968,018, chr16.73,100,688, chr5.174,115,531, chr5.1,831,958 and chr9.98,547,011) and the remaining two DMCs chr17.45,330,989 and chr14.105,353,824 were within in the integrin beta 3 (*ITGB3*) promoter and *KIAA0284* exon, respectively. *ITGB3* methylation was significantly decreased in the post-surgery (0.5 ± 0.5%) and lean controls (0 ± 0%) *versus* pre-surgery (13.6 ± 2.7%, *P* < 0.05). This decreased methylation post-surgery was associated with an increase in *ITGB3* gene expression (fold change + 1.52, *P* = 0.0087). In addition, we showed that *ITGB3* promoter methylation in vitro significantly suppressed transcriptional activity (*P* < 0.05). Transcription factor binding analysis for *ITGB3* chr17.45,330,989 identified three putative transcription factor binding motifs; PAX-5, p53 and AP-2alphaA.

**Conclusions:**

These results demonstrate that weight loss after RYGB alters the epigenome through DNA methylation. In particular, this study highlights *ITGB3* as a novel gene that may contribute to the metabolic improvements observed post-surgery. Future additional studies are warranted to address the exact mechanism of *ITGB3* in skeletal muscle.

**Supplementary Information:**

The online version contains supplementary material available at 10.1186/s13148-021-01086-6.

## Background

Obesity is a chronic and complex metabolic disease. Over the past 50 years, the prevalence of obesity has increased worldwide to epidemic proportions [1]. According to the World Health Organization (WHO), 39% of adults were overweight in 2016, and 13% were obese. In the USA, the age-adjusted prevalence of obesity in adults was 42.4% [2]. Obesity is a major health challenge and increases the risk of a number of chronic diseases including type 2 diabetes, hypertension, dyslipidemia and cardiovascular disease [3]. People who are obese often present with hyperinsulinemia and an underlying insulin resistance, which increases their risk for type 2 diabetes [4]. For many patients, lifestyle modifications such as diet and exercise and pharmacological therapies are ineffective at achieving long-term weight loss. Another alternative for obesity treatment is bariatric surgery. Bariatric surgery is a weight loss treatment for patients with a BMI of greater than or equal to 35 kg/m^2^ along with underlying comorbidities or with a body mass index (BMI) greater than or equal to 40 kg/m^2^. We [5] and others [6–13] showed that bariatric surgery results in weight loss along with metabolic improvements. The metabolic benefits of surgical intervention include improved blood glucose levels and improvements in insulin sensitivity and secretion [5, 11–13]. In addition, a meta-analysis across 621 studies with 135,246 patients revealed that type 2 diabetes is resolved in the greater majority of patients following bariatric surgery [14].

The molecular mechanism by which insulin exerts its actions in skeletal muscle has led to a greater and detailed understanding of insulin signaling and glucose transport [15–19]. However, the mechanisms to explain the metabolic improvements following weight loss induced by bariatric surgery in skeletal muscle are less known. We previously showed a global analysis of protein abundance changes in combination with transcriptomic analyses in skeletal muscle following Roux-en-Y gastric bypass (RYGB) surgery [5]. In that study, we showed that bariatric surgery results in skeletal muscle changes for genes and proteins involved in insulin signaling and ribosomal pathways [5]. We also showed that the cytoskeletal interacting protein sorbin and SH3 domain containing 3 (*SORBS3*) was decreased in methylation in the skeletal muscle of patients following weight loss induced by RYGB [20]. Gastaldi et al. showed that weight loss induced by RYGB resulted in an upregulation of the peroxisome proliferator-activated receptor gamma coactivator (*PPARGC1A*) gene [21]. In another study, it was demonstrated that insulin signaling genes were increased in skeletal muscle following bariatric surgery [22]. Barres et al. showed that obesity was associated with altered expression of a number of genes coding for metabolic and mitochondrial proteins [11]. In that same study, they showed that surgery-induced weight loss resulted in a normalization of those genes to levels observed in healthy, weight controls [11].

The effects of bariatric surgery on the epigenome of skeletal muscle is less known. To our knowledge, there has been only one study that investigated global methylation changes in patients before and after bariatric surgery. The study of Barres et al. showed that global methylation in human skeletal muscle is unaltered by either obesity or RYGB surgery-induced weight loss [11]. However, in that same study, the authors performed promoter-specific DNA methylation of a subset of metabolic genes. Among the 14 metabolic genes analyzed, promoter methylation of 11 genes including *PPARGC1A* and pyruvate dehydrogenase kinase 4 (*PDK4*) were normalized to levels observed in the healthy weight controls [11]. The aim of the present study was to determine the global DNA methylation changes that occurred in skeletal muscle following RYGB. RYGB is one of the most common surgeries performed to treat obesity and combines restrictive and malabsorptive techniques. The focus of this study was on the epigenetic marker DNA methylation, because the addition or removal of the methyl marks may influence and regulate gene expression. For this study, we hypothesized that bariatric surgery would alter the skeletal muscle epigenome of genes involved in insulin signaling and ribosomal pathways, in part because our previous transcriptomic and proteomic analyses revealed these changes following bariatric surgery [5]. Our global methylation analysis approach allowed us to test this specific hypothesis while at the same time performing a genomewide approach that revealed novel and unanticipated findings.

## Results

### Participants

Table 1 shows the anthropometric and clinical characteristics for the RYGB participants post- *versus* pre-surgery and the lean healthy controls. The metabolic data for the RYGB participants post- *versus* pre-surgery have been described in a previous publication [5]. Briefly, 3-month post-surgery, significant improvements were observed in BMI, body fat percentage, cholesterol, low-density lipoprotein (LDL), fasting plasma glucose (FPG) and fasting serum insulin (FSI) (Table 1). There were no significant improvements in blood pressure, triglycerides, high-density lipoprotein (HDL), hemoglobin A1c (HbA1c) and insulin-stimulated glucose disposal (M-value) following RYGB surgery (Table 1). As expected, the lean controls had significantly lower BMI, body fat percentage and HbA1c compared to the pre-surgery participants. The lean controls showed significantly higher levels of insulin-stimulated glucose disposal and HDL compared to the pre-surgery participants. The FSI post-surgery was normalized to levels similar to those observed in lean controls (Table 1).

**Table 1:** Participant characteristics

	Lean	Pre-surgery obese	Post-surgery obese	*P* value versus Lean Pre	*P* value Pre versus Post	*P* value Lean versus Post
Sex	Four female	Seven female				
Age (years)	38.5 ± 5.8	45.1 ± 3.6	45.3 ± 3.5	NS	NS	NS
Body mass index (kg/m^2^)	23.2 ± 0.9	42.1 ± 2.2	35.3 ± 1.8	< 0.001	< 0.001	< 0.01
Body fat (%)	30.3 ± 1.0	46.4 ± 1.2	40.6 ± 1.3	< 0.00001	< 0.01	< 0.001
Waist circumference (cm)	83.5 ± 3.2	122.7 ± 6.3	109.4 ± 109.4	< 0.01	< 0.0001	< 0.05
Systolic blood pressure (mmHg)	102.3 ± 12.6	125.1 ± 3.9	119.1 ± 4.6	NS	NS	NS
Diastolic blood pressure (mmHg)	59.8 ± 13.3	71.7 ± 2.0	75.1 ± 1.7	NS	NS	NS
Triglycerides (mg/dL)	92.8 ± 7.8	121.9 ± 17.5	107.7 ± 11.2	NS	NS	NS
Cholesterol (mg/dL)	202.3 ± 11.4	181.4 ± 13.2	151.5 ± 11.2	NS	< 0.01	< 0.05
High-density lipoprotein (mg/dL)	63.0 ± 9.0	45.0 ± 2.7	45.0 ± 2.5	< 0.05	NS	< 0.05
Low-density lipoprotein (mg/dL)	120.8 ± 7.6	112.1 ± 11.9	84.8 ± 10.5	NS	< 0.01	< 0.05
Hemoglobin A1c %	5.3 ± 0.1	6.0 ± 0.2	5.7 ± 0.1	< 0.05	NS	< 0.05
Fasting plasma glucose (mg/dL)	87.2 ± 3.1	104.2 ± 7.8	86.7 ± 3.1	NS	< 0.05	NS
Fasting serum insulin (uIU/mL)	7.9 ± 4.2	18.2 ± 2.7	7.5 ± 1.0	< 0.01	< 0.01	NS
M-value (mg/kg/min)	6.7 ± 0.7	2.4 ± 0.3	2.9 ± 0.4	< 0.001	NS	< 0.001

Data are mean ± SEM

### Methylation sites distribution

**Fig. 1:**
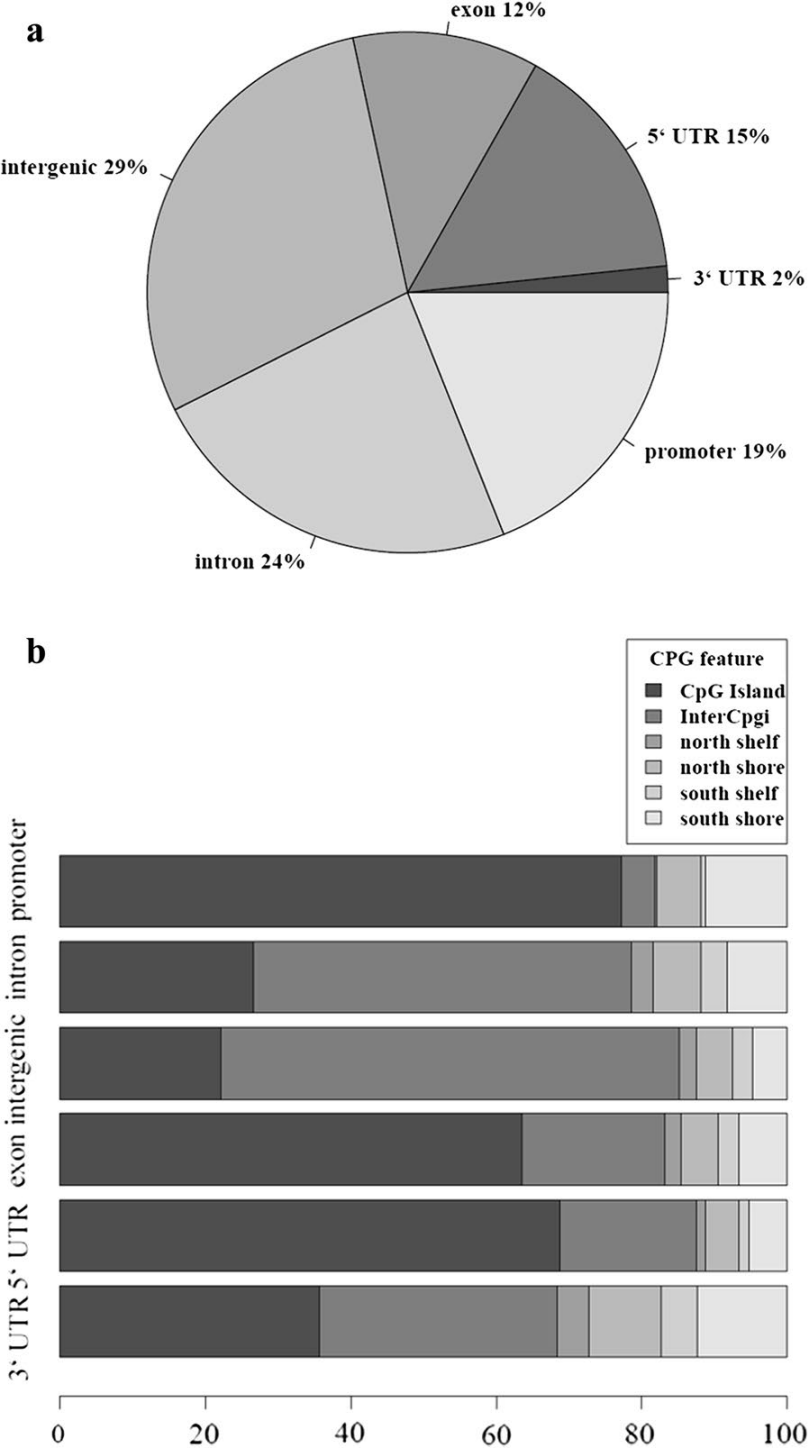
Methylation sites captured were mapped in the context of gene regions (**a**) and CpG island features (**b**). The regions were defined using UCSC browser refGene and CpG island tracks. The promoter region was defined as 1000 bp (basepairs) upstream of the transcription start site (TSS); untranslated region (UTR); CpG island is 200–3000 bp stretch of DNA with a C + G content of 50% and observed CpG/expected CpG in excess of 0.6; North (N) and South (S) shores flank the CpG island by 0–2000 bp; the North (N) and South (S) shelf flank the shores by 2000 bp (2000–4000 bp from the island)

**Fig. 2:**
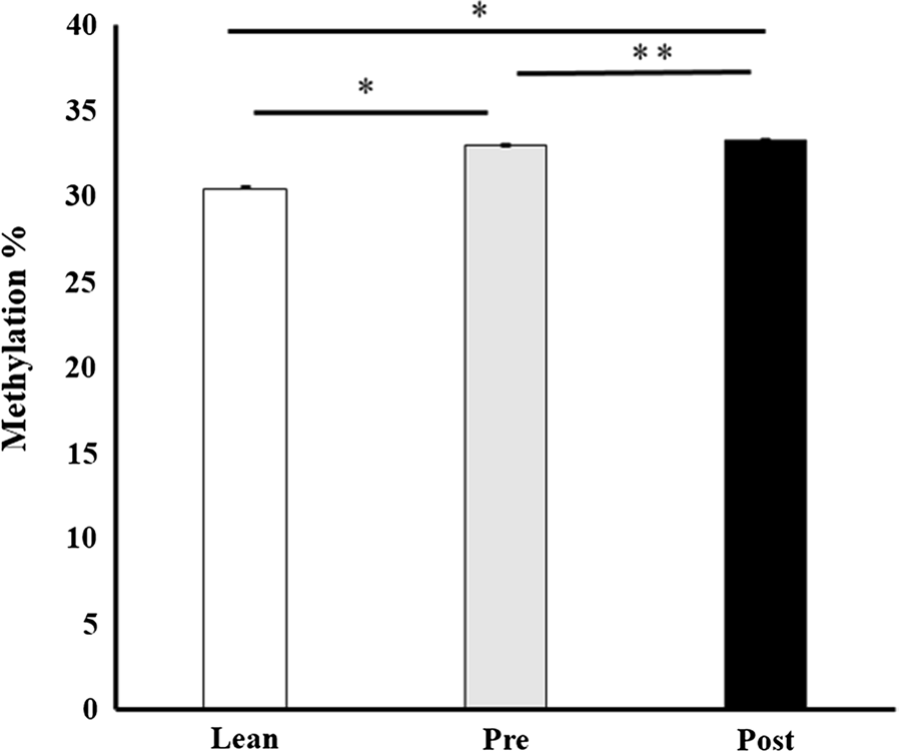
Reduced representation bisulfite sequencing analysis of global DNA methylation of DNA extracted from muscle in lean participants and patients pre- *versus* post-surgery. Data are mean ± SEM. *P < 0.05 using Mann–Whitney U test. **P < 0.05 using Wilcoxon signed-rank test

**Fig. 3:**
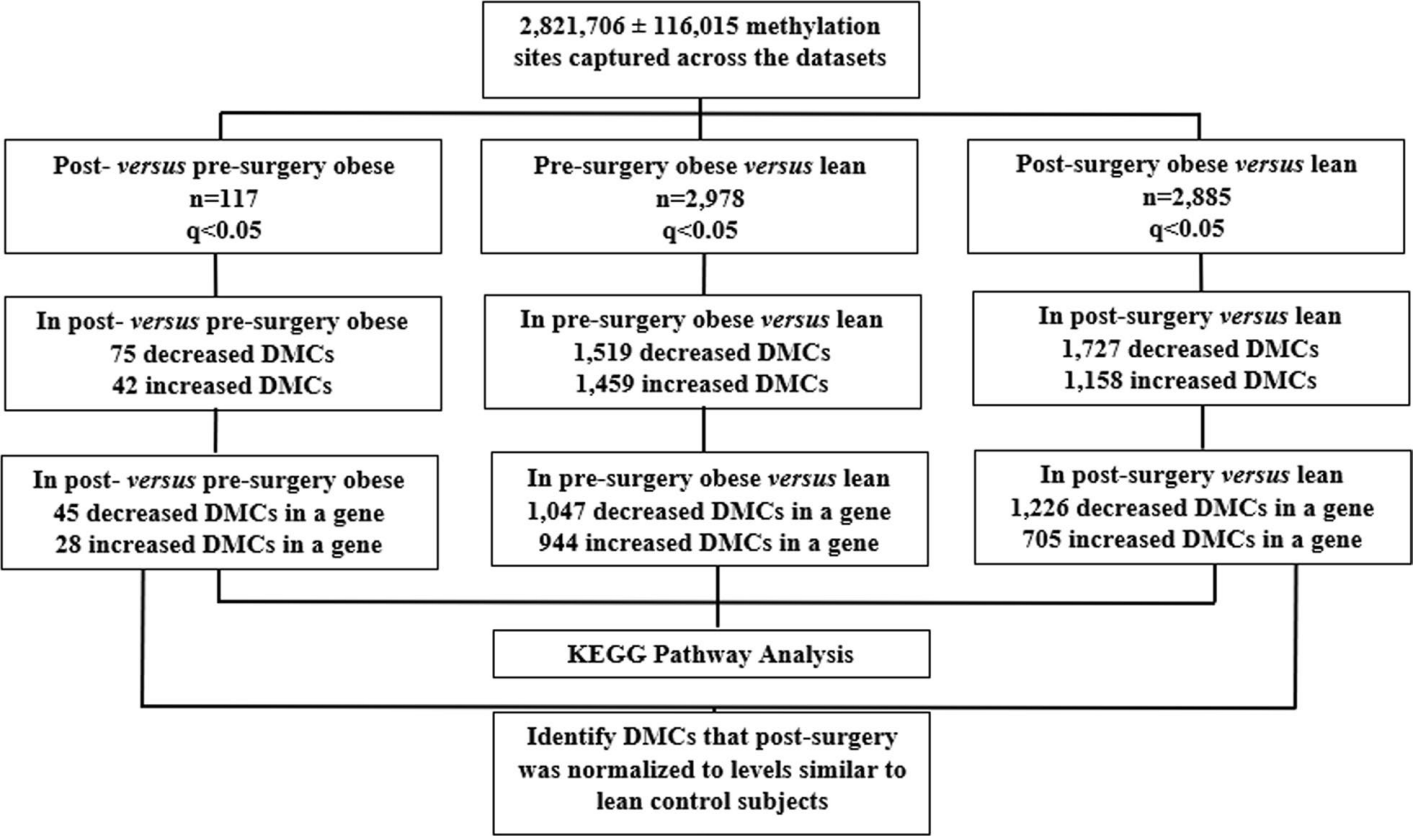
Workflow of methylation analysis across the lean, pre-surgery obese and post-surgery groups

Prior to the quality control of the sequence data, 5,003,481 sites were captured across the MethylSig data object for the lean, pre-surgery obese and post-surgery obese RRBS datasets. A threshold of greater than 80% call rate and a minimum of 5X coverage for the RRBS data was set. In total, 2,821,706 ± 116,015 methylation sites were captured across the methylation datasets when this threshold was set. The distribution of the methylation sites across the three datasets was defined by genic regions (Fig. 1a) and CpG Island features (Fig. 1b). The majority of the methylation sites were in intergenic (29%), intronic (24%) and promoter (19%) regions (Fig. 1a). Sites in the promoter and 5′ untranslated regions (UTR) dominantly overlapped with CpG islands (Fig. 1b). As shown in Fig. 2, global methylation was significantly higher post- *versus* pre-surgery (33.31 ± 0.02% *versus* 32.97 ± 0.02%, *P* < 0.05). In addition, global methylation was significantly lower in the lean participants (30.46 ± 0.02%) compared with post- and pre-surgery (*P* < 0.05). A summary of the methylation analysis findings is shown in Fig. 3.

### Post- versus pre-surgery methylation analyses

MethylSig analysis revealed 117 differentially methylated cytosines (DMCs) that were significantly altered in methylation post-surgery *versus* pre-surgery (Benjamini–Hochberg *q* < 0.05) Of the 117 DMCs, 75 were decreased and 42 were increased post-surgery. Of the decreasers and increasers, 45 and 28 were assigned to a gene, respectively. The remaining DMCs were not associated with a gene. Table 2 shows the 45 genes with significant (Benjamini–Hochberg *q* < 0.05) DMCs that were decreased post- *versus* pre-surgery. Table 3 shows the 28 genes with significant (Benjamini–Hochberg *q* < 0.05) DMCs that were increased post-surgery *versus* pre-surgery.

**Table 2:** Differentially methylated cytosines (DMCs; *q* < 0.05) decreased post-surgery versus pre-surgery

Chr	Position	Gene	Pre-surgery methylation %	Post-surgery methylation %	*P* value	*q* value	Genic region	CpG region
chr19	9,271,227	ZNF317	99.54 ± 0.46	88.87 ± 2.27	8.86E−09	0.0020	Exon	CpG_Island
chrX	41,782,532	CASK	36.44 ± 4.47	7.49 ± 1.49	1.61E−08	0.0028	Promoter	CpG_Island
chr9	134,346,329	PRRC2B	99.15 ± 0.57	86.92 ± 2.80	1.69E−08	0.0028	Exon	InterCpgi
chr15	101,420,903	ALDH1A3	81.98 ± 2.87	58.77 ± 5.28	3.90E−08	0.0050	Intron	CpG_Island
chr3	13,696,951	LOC285375	9.02 ± 2.58	0 ± 0	3.63E−08	0.0050	Intergenic	CpG_Island
chr11	70,008,217	ANO1	99.47 ± 0.53	82.21 ± 2.68	6.57E−08	0.0079	Intron	InterCpgi
chr5	9,509,512	SEMA5A	100 ± 0	91.29 ± 2.45	8.58E−08	0.0091	utr5	InterCpgi
chr19	48,824,452 ≠	CCDC114	79.96 ± 2.47	52.67 ± 3.92	1.11E−07	0.0105	Promoter	North_shore
chr19	48,824,452 ≠	EMP3	79.96 ± 2.47	52.67 ± 3.92	1.11E−07	0.0105	Promoter	North_shore
chr15	76,634,525	ISL2	6.79 ± 1.30	0 ± 0	1.09E−07	0.0105	utr3	CpG_Island
chr16	1,774,262	MAPK8IP3	96.07 ± 0.89	86.94 ± 2.61	1.36E−07	0.0116	Intron	InterCpgi
chr15	62,457,572	C2CD4B	52.02 ± 4.77	15.90 ± 4.03	1.48E−07	0.0122	Promoter	CpG_Island
chr16	89,687,436	DPEP1	74.63 ± 2.21	46.44 ± 4.95	1.80E−07	0.0135	utr5	InterCpgi
chr17	45,330,989	ITGB3	13.64 ± 2.74	0.51 ± 0.51	1.96E−07	0.0135	Promoter	CpG_Island
chr9	104,075,142	LPPR1	100 ± 0	82.89 ± 5.22	2.13E−07	0.0135	Exon	InterCpgi
chr16	30,990,627	SETD1A	86.89 ± 3.56	57.56 ± 5.29	1.84E−07	0.0135	Exon	North_shore
chr12	54,345,965	HOXC12	15.68 ± 1.11	3.11 ± 1.14	2.69E−07	0.0159	Intergenic	South_shore
chr19	619,189	POLRMT	99.40 ± 0.6	91.17 ± 1.00	3.34E−07	0.0188	Intron	CpG_Island
chr11	628,172^‡^	CDHR5	100 ± 0	89.48 ± 2.13	3.74E−07	0.0192	Promoter	South_shore
chr11	628,172^‡^	SCT	100 ± 0	89.48 ± 2.13	3.74E−07	0.0192	Promoter	South_shore
chr10	88,269,670	WAPAL	99.68 ± 0.32	91.58 ± 1.90	3.76E−07	0.0192	Intron	InterCpgi
chr1	235,979,239	LYST	42.96 ± 5.36	15.09 ± 3.25	3.94E−07	0.0197	Intron	InterCpgi
chr19	50,098,039	PRR12	87.92 ± 1.83	67.43 ± 3.66	4.39E−07	0.0216	Exon	CpG_Island
chr8	101,723,031	PABPC1	83.96 ± 3.70	42.12 ± 5.68	4.62E−07	0.0219	Intron	InterCpgi
chr15	23,932,055	NDN	39.77 ± 5.54	14.89 ± 3.47	4.81E−07	0.0222	Exon	CpG_Island
chr5	1,491,652	LPCAT1	98.22 ± 1.19	79.85 ± 3.10	5.04E−07	0.0226	Intron	South_shore
chr11	61,486,429	DAGLA	58.08 ± 3.83	34.62 ± 3.51	5.53E−07	0.0241	utr5	North_shore
chr3	126,746,778	PLXNA1	100 ± 0	87.57 ± 1.95	5.87E−07	0.0251	Intron	North_shore
chr15	23,892,609	MAGEL2	54.77 ± 8.17	19.70 ± 3.99	6.66E−07	0.0276	Exon	InterCpgi
chr4	111,553,834	PITX2	88.67 ± 3.28	55.56 ± 9.64	7.47E−07	0.0297	Intron	CpG_Island
chr17	79,497,291	FSCN2	57.24 ± 5.14	29.65 ± 3.61	8.94E−07	0.0308	Intron	South_shore
chr14	24,666,733	TM9SF1	93.83 ± 2.06	71.93 ± 3.85	8.75E−07	0.0308	Promoter	South_shore
chr21	43,859,102	UBASH3A	86.97 ± 2.24	68.44 ± 4.45	8.62E−07	0.0308	Intron	InterCpgi
chrX	152,600,028	ZNF275	48.08 ± 2.35	25.34 ± 3.90	8.31E−07	0.0308	utr5	CpG_Island
chr4	3,678,810	LOC100133461	93.87 ± 2.40	63.82 ± 6.14	9.43E−07	0.0317	Intergenic	InterCpgi
chr13	67,135,465	PCDH9	98.97 ± 0.50	89.39 ± 1.64	1.14E−06	0.0370	Intron	InterCpgi
chrX	10,188,915	CLCN4	100 ± 0	91.75 ± 2.13	1.42E−06	0.0419	Exon	InterCpgi
chr18	34,274,365	FHOD3	59.66 ± 4.19	34.53 ± 3.20	1.51E−06	0.0429	Intron	InterCpgi
chr20	62,329,849	TNFRSF6B	76.74 ± 2.63	57.78 ± 4.65	1.62E−06	0.0448	Exon	South_shore
chr15	101,421,027	ALDH1A3	79.81 ± 3.85	54.34 ± 5.33	2.04E−06	0.0479	Intron	CpG_Island
chr20	3,778,167	CDC25B	95.43 ± 1.99	75.05 ± 3.37	1.90E−06	0.0479	utr5	South_shore
chr7	27169037^^^	HOXA3	94.58 ± 1.54	78.97 ± 4.24	1.96E−06	0.0479	Exon	North_shore
chr7	27169037^^^	HOXA4	94.58 ± 1.54	78.97 ± 4.24	1.96E−06	0.0479	Exon	north_shore
chr6	100,906,424	SIM1	49.04 ± 2.93	18.82 ± 4.42	2.00E−06	0.0479	Intron	CpG_Island
chr22	19,747,159	TBX1	44.30 ± 4.95	22.56 ± 2.96	2.14E−06	0.0498	utr5	South_shore

Data are mean ± SEM. The *P* value is calculated from the MethylSig analysis. The *q* value is generated by Benjamini–Hochberg multiple testing correction

^‡^Two genes [SCT and CDHR5] are associated with the same differentially methylated cytosine [Chr11:628172]; ≠ Two genes [EMP3 and CCDC114] are associated with the same differentially methylated cytosine [Chr19: 48824452]

^≠^ Two genes [EMP3 and CCDC114] are associated with the same differentially methylated cytosine [Chr19: 48824452]

^^^Two genes [HOXA3 and HOXA4] are associated with the same differentially methylated cytosine [Chr7: 27169037]

**Table 3:** Differentially methylated cytosines (DMCs; *q* < 0.05) that were increased post-surgery *versus* pre-surgery

Chr	Position	Gene	Pre-surgery methylation %	Post-surgery methylation %	*P* value	*q* value	Genic region	CpG region
chr2	27,498,318	DNAJC5G	51.83 ± 9.21	89.02 ± 2.98	1.01E−09	0.00067	utr5	InterCpgi
chr7	20,830,624	SP8	1.30 ± 0.84	13.90 ± 1.02	2.01E−09	0.00076	Intergenic	CpG_Island
chr1	228,504,058	OBSCN	42.29 ± 6.19	84.61 ± 2.99	3.28E−09	0.00109	Intron	CpG_Island
chr10	119,271,421	EMX2OS	42.57 ± 2.47	68.77 ± 3.23	6.08E−09	0.00161	Intergenic	InterCpgi
chr1	51,154,377	FAF1	92.12 ± 2.38	100 ± 0	1.16E−08	0.00220	Intron	InterCpgi
chr2	11,925,218	LPIN1	0 ± 0	9.68 ± 1.23	1.55E−07	0.01243	Intron	CpG_Island
chr1	240,347,650	FMN2	84.75 ± 4.18	99.35 ± 0.65	2.06E−07	0.01352	Intron	InterCpgi
chr4	57,068,530	KIAA1211	83.59 ± 2.29	98.25 ± 1.13	1.89E−07	0.01352	utr5	InterCpgi
chr18	13,431,993	C18orf1	84.39 ± 3.53	100 ± 0	3.16E−07	0.01820	Intron	InterCpgi
chr3	113,160,431	WDR52	13.71 ± 2.73	46.89 ± 6.19	3.49E−07	0.01886	Promoter	CpG_Island
chr13	96,414,410	DNAJC3	90.20 ± 2.64	100 ± 0	5.33E−07	0.02357	Intron	InterCpgi
chr1	197,108,554	ASPM	86.50 ± 4.35	100 ± 0	7.84E−07	0.03038	Intron	InterCpgi
chr17	5,462,805	NLRP1	80.80 ± 5.66	99.05 ± 0.95	7.90E−07	0.03038	Exon	InterCpgi
chr19	54,872,598	LAIR1	67.12 ± 6.27	93.87 ± 2.11	8.60E−07	0.03079	Exon	InterCpgi
chr11	36,512,043	TRAF6	40.53 ± 4.68	65.66 ± 3.25	8.80E−07	0.03079	Exon	InterCpgi
chr15	52,248,473	LEO1	18.60 ± 3.51	45.53 ± 4.24	9.21E−07	0.03131	Intron	InterCpgi
chr22	50,919,564	ADM2	2.23 ± 0.81	11.87 ± 1.89	1.17E−06	0.03742	Promoter	CpG_Island
chr7	135,258,846	NUP205	88.18 ± 3.66	100 ± 0	1.20E−06	0.03793	Intron	InterCpgi
chr1	93,172,465	EVI5	83.13 ± 3.46	100 ± 0	1.42E−06	0.04189	Intron	InterCpgi
chr12	133,125,172	FBRSL1	88.19 ± 3.33	100 ± 0	1.44E−06	0.04203	intron	North_shore
chr5	137,729,043	KDM3B	84.36 ± 3.76	96.62 ± 1.45	1.52E−06	0.04294	Exon	InterCpgi
chr3	9,989,586	PRRT3	45.47 ± 3.64	74.18 ± 3.58	1.49E−06	0.04294	Exon	CpG_Island
chrX	66,764,054	AR	15.74 ± 4.49	42.72 ± 2.58	1.67E−06	0.04508	utr5	CpG_Island
chr1	91,184,127	BARHL2	0 ± 0	2.97 ± 0.83	1.81E−06	0.04740	Promoter	CpG_Island
chr7	939,123	ADAP1	40.13 ± 9.99	74.26 ± 3.10	1.83E−06	0.04759	Exon	South_shelf
chr1	34,642,648	C1orf94	0 ± 0	7.35 ± 2.25	2.04E−06	0.04785	utr5	CpG_Island
chr7	71,562,951	CALN1	81.90 ± 4.23	98.70 ± 1.30	1.96E−06	0.04785	Intron	InterCpgi
chr14	105,353,824	KIAA0284	62.07 ± 7.36	90.15 ± 4.41	1.93E−06	0.04785	Exon	South_shelf

Data are mean ± SEM. The *P* value is calculated from the MethylSig analysis. The *q* value is generated by Benjamini–Hochberg multiple testing correction

There were no KEGG pathways enriched in either the decreased or increased DMC genes in the post- *versus* pre-surgery analyses. DAVID gene ontology analysis of the DMC genes that was decreased in the post-surgery group revealed an enrichment in 11 functional categories (Additional file 1). The top two significant functional groups were DNA binding (*TNFRSF6B, ZNF275, NDN, PRR12, TBX1, POLRMT, ISL2, HOXC12, HOXA3, HOXA4, ZNF317, SIM1* and *PITX2*) and regulation of RNA metabolic process (*ZNF275, ISL2, HOXC12, HOXA3, HOXA4, NDN, ZNF317, CASK, TBX1, PABPC1, SIM1* and *PITX2*). Moreover, Additional file 1 shows a number of developmental functional groups identified including skeletal system morphogenesis and embryonic skeletal system development/morphogenesis. DAVID gene ontology analysis on the genes with increased DMCs post-surgery identified 11 enriched groups (Additional file 2). The heat shock protein binding (*FAF1, DNAJC5G* and *DNAJC3*) was the most significantly enriched group. Interestingly, the increased DMC genes *OBSCN, AR, FAF1, TRAF6* and *NLRP1* grouped together in functional groups related to positive regulation of cell death and positive regulation of apoptosis (Additional file 2).

### Pre-surgery obese versus lean methylation analyses

MethylSig analysis revealed 2,978 differentially methylated sites (DMCs) that were significantly altered in the pre-surgery obese *versus* the lean controls (Benjamini–Hochberg *q* < 0.05) (Additional file 3). Of the 2,978 DMCs, 1,519 were decreased in methylation in the pre-surgery obese group compared to the lean controls (1047 DMCs were assigned to a gene, Additional file 3). In addition, 1,459 DMCs were increased in methylation in the pre-surgery obese *versus* the lean group (944 DMCs were assigned to a gene, Additional file 3).

KEGG pathway analysis of the DMC genes that were decreased in the pre-surgery obese group revealed an enrichment in 11 pathways (Additional file 4), including regulation of actin cytoskeleton (hsa04810), focal adhesion (hsa04510), insulin signaling pathway (hsa04910) and adipocytokine signaling pathway (hsa04920). There were 6 KEGG pathways enriched in the increased DMC genes in the pre-surgery obese group including long-term potentiation (hsa04720), phosphatidylinositol signaling system (hsa04070), chemokine signaling pathway (hsa04062) and calcium signaling pathway (hsa04020). Interestingly, the focal adhesion pathway (hsa04510) was also enriched in the increased DMC genes (Additional file 5).

### Post-surgery obese versus lean methylation analyses

MethylSig analysis revealed 2,885 differentially methylated sites (DMCs) that were significantly altered in the post-surgery obese *versus* the lean controls (Benjamini–Hochberg *q* < 0.05) (Additional file 6). Of the 2,885 DMCs, 1,727 were decreased in methylation in the post-surgery obese group compared to the lean controls (1,226 DMCs were assigned to a gene, Additional file 6). In addition, 1,158 DMCs were increased in methylation in the post-surgery obese *versus* the lean group (705 DMCs were assigned to a gene, Additional file 6).

KEGG pathway analysis of the DMC genes that were decreased in the post-surgery obese group revealed an enrichment in 24 pathways (Additional file 7), including regulation of actin cytoskeleton (hsa04810), focal adhesion (hsa04510), ECM-receptor interaction (hsa04512), PI3K-Akt signaling pathway (hsa04151), insulin resistance (hsa04931), adipocytokine signaling pathway (hsa04920), MAPK signaling pathway (hsa04010), insulin secretion (hsa04911), insulin signaling pathway (hsa04910) and AMPK signaling pathway (hsa04152). There were 6 KEGG pathways enriched in the increased DMC genes in the post-surgery obese group including calcium signaling pathway (hsa04020), neuroactive ligand-receptor interaction (hsa04080), notch signaling pathway (hsa04330) and mucin type O-glycan biosynthesis (hsa00512) (Additional file 8).

### Post-surgery DMCs normalized to lean control methylation analyses

**Fig. 4:**
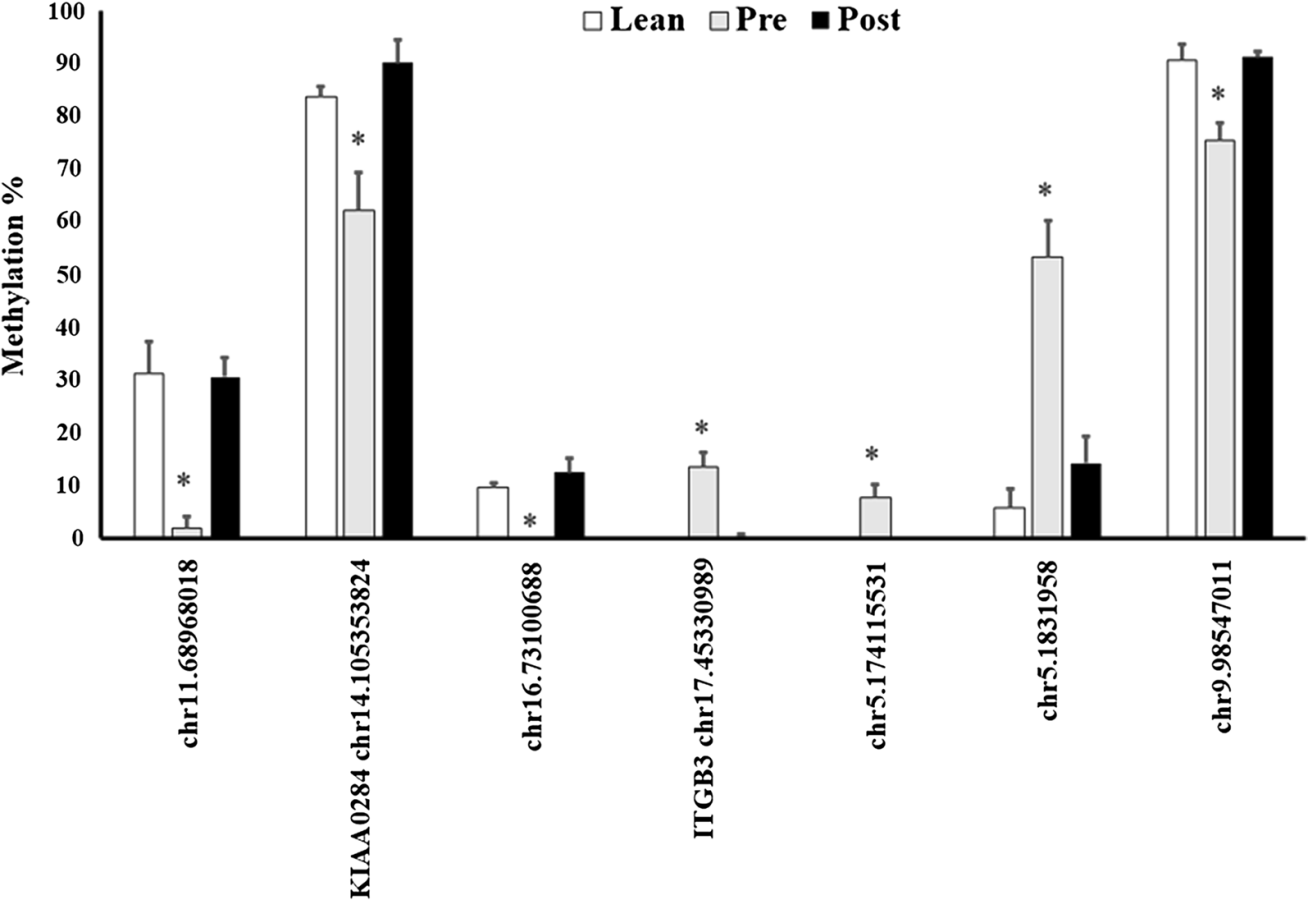
Differentially methylation cytosines that post-surgery were normalized to levels similar to those observed in the lean control subjects. Data are mean ± SEM. *Benjamini–Hochberg *q* < 0.05

We identified 7 DMCs that post-surgery were normalized to levels similar to those observed in the lean control subjects (Fig. 4). In order to be considered normalized, we stipulated that DMCs had to be significantly (Benjamini–Hochberg *q* < 0.05) different in both the pre-surgery obese *versus* lean and post- *versus* pre-surgery analyses. Of the 7 DMCs, five were in intergenic regions at positions chr11.68,968,018, chr16.73,100,688, chr5.174,115,531, chr5.1,831,958 and chr9.98,547,011. The remaining two DMCs chr17.45,330,989 and chr14.105,353,824 were associated with *ITGB3* and *KIAA0284* gene, respectively. The *ITGB3* DMC was within a promoter region and *KIAA0284* DMC was in an exonic region.

### ITGB3 gene expression in post- versus pre-surgery

The quantitative RT-PCR results showed an increase in gene expression of *ITGB3* post-surgery compared to pre-surgery (+ 1.52 versus 1.0-fold change, respectively; *P* = 0.0087). Skeletal muscle RNA was not available to perform the gene expression analysis on the lean participants.

### ITGB3 promoter methylation in vitro alters reporter gene expression

**Fig. 5:**
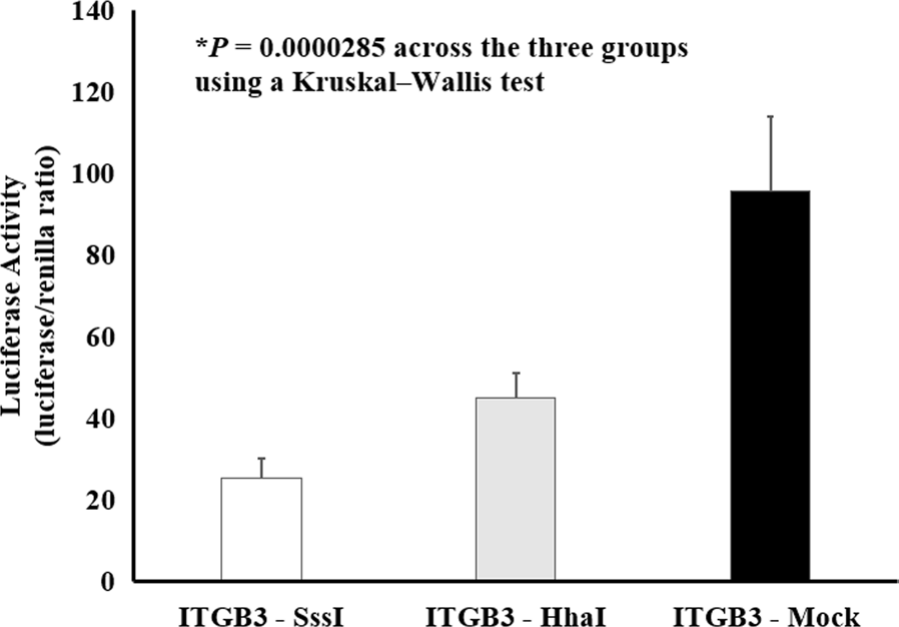
In vitro DNA methylation of the *ITGB3* human promoter is associated with decreased gene expression. Data are mean ± SEM. The mean represents 3 independent experiments with 5 replicates per experiment

The *ITGB3* construct was created to test the effect of DNA methylation on transcriptional activity. The level of suppressed transcriptional activity, as measured by luciferase activity, was determined in comparison to the mock methylated control (Fig. 5). As shown in Fig. 5, when the *ITGB3* construct was methylated in vitro using the HhaI enzyme (GCGC, *n* = 6 sites), transcriptional activity was suppressed by 52.8%. Moreover, when the *ITGB3* construct was methylated in vitro using the SssI enzyme (CG, *n* = 53 sites), transcriptional activity was suppressed by 73.4%. There was a statistically significant difference between the groups as determined by a Kruskal–Wallis test (Chi square = 20.931, *P* = 0.0000285, df = 2).

### Predicted transcription factor binding analysis for ITGB3 chr17.45,330,989

PROMO analysis identified three potential transcription factor binding motifs overlapping the differentially methylated cytosine (chr17.45,330,989) of *ITGB3*. The transcription factors were PAX-5, p53 and AP-2alphaA.

## Discussion

The overarching goal of the present study was to determine whether global DNA methylation changes occur in skeletal muscle following Roux-en-Y gastric bypass (RYGB). We previously published the metabolic changes observed in these patients post- *versus* pre-surgery [5]; therefore, the focus of this paper was on the epigenetic changes that occur with bariatric surgery. As part of this study, we were able to capture overall global methylation post- *versus* pre-surgery and also identify novel differentially methylated cytosines. We included a lean control group, in part to identify differentially methylated cytosines (DMCs) that were normalized post-surgery to the lean group. By including the lean control group, we were able to identify DMCs that were altered in the pre-surgery obese group compared to the lean.

In this study, we predicted that global methylation across the groups would be unchanged. This was in part, because Barres et al. showed that global methylation levels were similar before and after RYGB surgery-induced weight loss, as well as being similar between the obese and nonobese women [11]. We showed that the post-surgery group had the highest global methylation at 33.31 ± 0.02% compared to the pre-surgery at 32.97 ± 0.02% and the lean group at 30.46 ± 0.02%. Interestingly, even though the post-surgery group had an intervention that resulted in weight loss and improvements in cholesterol, LDL, FPG and FSI, the global methylation average was significantly higher than the lean participants. In fact, the post-surgery group had a slightly higher average global methylation compared to the pre-surgery. The difference between the post- *versus* pre-surgery global methylation average was a change of 0.34. Although this was a significant change, we consider the individual DMC data that is changing within these groups to be more biologically relevant than the global averages. We observed a much greater number of DMCs that were altered when comparing the pre-surgery obese to the lean control group (*n* = 2978), and the post-surgery obese to the lean control group (*n* = 2885). With the post- *versus* pre-surgery analysis, we identified a much lower number of DMCs (*n* = 117). This finding alone suggests that the methylation changes are much more pronounced between the obese and lean phenotypes compared to the surgery intervention.

To our knowledge, this is one of the first studies to examine global differential DNA methylation in skeletal muscle following RYGB surgery. A study by Barres et al. measured skeletal muscle global CpG and non-CpG methylation in patients before and 6 months after bariatric surgery, and in lean healthy controls [11]. In that study, they showed that global methylation was unaltered by obesity or RYGB surgery. As part of that same study, they measured the promoter methylation of 14 metabolic genes and showed that 11 genes post-surgery including *PPARGC1* and *PDK1* were normalized to levels observed in the lean control group [11]. In the present study, we identified 7 DMCs (two DMCs associated with *ITGB3* and *KIAA0284*) that were normalized post-surgery to lean controls. The genes that were normalized in the Barres et al. study were not in common with this study [11]. This could be due to the time of the intervention, in the Barres study the *vastus lateralis* was collected 6-month post-surgery while we collected the biopsy at 3 months [11]. The discrepancy in the normalized sites in the genes may be explained by the different technologies used for measuring methylation.

Our analyses identified *ITGB3* as a potential gene that was altered in methylation and gene expression following weight loss induced by RYGB surgery. We showed that a differentially methylated cytosine in *ITGB3* was normalized post-surgery to levels observed in the lean healthy controls, even though the mean BMI between the lean and post-surgery groups were not comparable. The DMC was located in the promoter region of *ITGB3*, and typically sites in regulatory regions can impact gene expression. We showed that the decreased methylation at cytosine chr17.45,330,989 in *ITGB3* corresponded with an increase in gene expression post-surgery. The link between DNA methylation and gene expression changes were further established by in vitro measures using a luciferase assay. These results provide evidence for the regulatory role of promoter methylation on gene expression. We showed a stepwise decrease in luciferase expression with an increasing number of methylated sites. We observed decreased gene expression with both the methylation set by HhaI (GCGC, *n* = 6 sites) and SssI (CG, *n* = 53 sites). This suggests that methylation at multiple cytosines in the 1319 bp fragment of the human *ITGB3* promoter (chr17:45,329,911–45,331,229) is important.

We attempted to validate *ITGB3* DMC (chr17.45,330,989) using pyrosequencing (data not shown). The *ITGB3* DMC (chr17.45,330,989) site of interest resides in a homopolymer T-rich region. Specifically, there are two homopolymer T-regions prior to our site of interest. These homopolymer T-regions can be a challenge for pyrosequencing experiments as discussed by Fakruddin and Chowdhury (Am J Biochem Biotechnol. 8 (1): 14–20, 2012).   The results of the pyrosequencing experiments were questionable, in part, because of the dispensation of extra T’s in the sequence. We speculate that this was due to the homopolymer T-regions that lie in that region. Although we do not show validation with pyrosequencing, we were able to show that *ITGB3* gene expression was altered following bariatric surgery. Moreover, we showed that the *ITGB3* promoter methylation in vitro significantly suppressed transcriptional activity. These additional experiments to our RRBS finding suggest that *ITGB3* may be important in skeletal muscle.

In the present study, the exact mechanisms by which DNA methylation regulates the transcription of *ITGB3* was not investigated. We did perform transcription factor binding analysis of the *ITGB3* DMC chr17.45,330,989 to identify potential transcription factor binding motifs that may be important for influencing transcriptional regulation at this region. Paired box 5 (PAX-5), p53 and activating enhancer-binding protein 2-alpha (AP-2alphaA) overlapped with the *ITGB3* site of interest. Transcription factor p53 is a critical protein involved in many aspects of human physiology [23]. A review by Kung and Murphy provided evidence that p53 is a key player in diabetes, and that activation of p53 was shown to exacerbate diabetic phenotypes [23]. The role of PAX-5 and AP-2alphaA in metabolism specifically in skeletal muscle tissue is less known. Future studies are needed to determine whether DNA methylation at the *ITGB3* region influences transcription factor binding and the regulation of gene expression. Moreover, additional studies are warranted to measure DNA methylation of the transcription factors themselves to determine if that impacts how they bind to the DNA, and thereby impacts expression.

Integrin subunit beta 3 (*ITGB3*) is part of the integrin family. Integrins are transmembrane heterodimeric glycoprotein receptors that link the extracellular matrix (ECM) to the intracellular cytoskeleton [24]. *ITGB3* is most commonly expressed as a surface adhesion protein on platelets. It is an integral part of platelet aggregation by acting as a receptor for fibrinogen, von Willebrand factor, and fibronectin [25]. We performed global methylation analysis on skeletal muscle DNA, and our most significant finding was a DMC (chr17.45,330,989) in *ITGB3*. Recent data from numerous sources suggest that *ITGB3* is important for muscle differentiation [26–28]. A recent study of the longissimus dorsi muscle of neonatal pigs showed that *ITGB3* is involved in myogenic differentiation [26]. In addition, a study in C2C12 myoblasts showed that *ITGB3* is upregulated during myogenic differentiation [27]. Moreover, a recent review of multiple transcriptome and methylome datasets in human skeletal muscle identified *ITGB3* as an important gene that was upregulated and hypomethylated following acute resistance exercise [28]. In the present study, we observed an increased gene expression and decreased methylation in *ITGB3* following bariatric surgery. Although exercise and bariatric surgery are different environmental exposures, it is interesting that there are similar findings with *ITGB3* methylation and expression across the studies [28]. Liu et al. showed that *ITGB3* is a mediator of satellite cell differentiation in regenerating muscle [27]. Skeletal muscle satellite cells play a crucial role in muscle fiber maintenance, repair and remodeling. We speculate that the *ITGB3* increased gene expression may be due to the bariatric surgery having an impact on the regulation of satellite cells in human skeletal muscle. However, our study is preliminary and future studies are warranted to address the exact mechanism that *ITGB3* plays on satellite/muscle cell repairing and remodeling of the skeletal muscle tissue. It could be argued that the methylation and gene expression changes observed post- *versus* pre-surgery in *ITGB3* is coming from platelets within the skeletal muscle tissue. However, we showed no change in platelets levels following the bariatric surgery (Post-surgery: 300 ± 34 versus pre-surgery: 301 ± 32 billion/L, *P* = NS). Moreover, platelets lack a nucleus and therefore do not contain nuclear DNA.

Although the focus of this paper was on the post-surgery DMCs that were normalized to levels observed in lean controls, there were several pathways from the pre-surgery obese *versus* lean, and the post-surgery obese *versus* lean analyses that warrant discussion. KEGG pathway analysis revealed an enrichment in differentially methylated cytosines within mechanosignaling and metabolic pathways for both the pre-surgery *versus* lean and post-surgery *versus* lean. These common pathways included actin cytoskeleton, focal adhesion, insulin signaling and adipocytokine signaling. Previous work from our laboratory [29–34] and others [35, 36] showed that the extracellular matrix (ECM) and the structure and function of muscle itself may be involved in the pathogenesis of insulin resistance, which is an underlying feature of obesity and type 2 diabetes. In the present study, the pre-surgery obese and post-surgery obese patients were insulin resistant compared to the lean participants, as determined by the euglycemic hyperinsulinemic clamp. Our overarching hypothesis for insulin resistant skeletal muscle is that mechanosensing and signaling of contractile forces from the ECM, to the cytoskeleton, and finally, the nucleus, is altered, leading to changes in the expression of metabolic and nuclear encoded mitochondrial genes [34]. This present study provides additional evidence to our hypothesis by identifying methylation changes in genes coding for pathways related to the ECM, cytoskeletal and metabolic genes in the pre-surgery obese and post-surgery obese compared to the lean controls.

In summary, our findings showed that there is a greater number of DNA methylation changes in skeletal muscle in the pre-surgery obese *versus* lean and post-surgery obese *versus* lean analyses, compared to the post-surgery *versus* pre-surgery. We observed seven DMCs that were normalized to the levels observed in lean, healthy controls. Only one of the seven DMCs was in a regulatory promoter region; therefore, we focused our downstream analyses on the *ITGB3* gene. We observed a decreased methylation post-surgery that corresponded with an increase in *ITGB3* gene expression. We also showed that *ITGB3* promoter methylation in vitro significantly suppressed transcriptional activity. We identified several transcription factor regulatory regions within the differentially methylated *ITGB3* region. However, further work is required to understand the exact mechanisms of DNA methylation on the transcriptional regulation of *ITGB3*.

There are limitations to this study that warrant a discussion. First, one major limitation was the small number of participants studied. Second, the study participants were exclusively female. Third, the limited size of the muscle biopsies meant we were unable to perform the quantitative real-time PCR experiment in the lean participants. Finally, we were unable to validate the *ITGB3* methylation finding  due to the homopolymer T-rich regions. Despite these limitations, we identified a number of genes that were differentially methylated in the pre-surgery obese *versus* lean, and post-surgery obese *versus* lean analyses to support our overarching mechanosignaling hypothesis in insulin resistant muscle. Moreover, we identified a potential gene, *ITGB3* that may play an important role in muscle following bariatric surgery. Additional studies for the future include studying a larger number of participants (both male and female participants) and include multiple time points for biopsy collection. Moreover, additional studies are warranted to address the exact mechanism of action of *ITGB3* in skeletal muscle metabolism.

## Methods

### Participants

Seven insulin-resistant obese (BMI > 40 kg/m^2^) female subjects (45.1 ± 3.6 years) were studied before and three months after RYGB. The metabolic data for these subjects were included in a previous publication [5]. Studies were approved by the Institutional Review Boards at the Mayo Clinic in Arizona and Arizona State University and were performed in the Clinical Studies Infusion Unit (CSIU) at Mayo Clinic in Arizona. After giving informed written consent, each volunteer underwent a medical history, physical examination, screening laboratory tests, a 75-g oral glucose tolerance test. On a different day, participants reported fasting to the CSIU to undergo an 80 mU/m^2^ euglycemic–hyperinsulinemic clamp with muscle biopsies collected before insulin infusion, as previously described [5, 37]. Three months post-RYGB the participants returned to the CSIU and a repeat euglycemic–hyperinsulinemic clamp with muscle biopsy was performed. The Roux-en-Y gastric bypass was performed at the Bariatric Surgery Program at the Mayo Clinic in Arizona [5]. Details of the program and the surgical procedures were described in our previous publication [5].

### Substrate and hormone determinations

The Biospecimens Accessioning and Processing Core at the Mayo Clinic in Arizona performed the screening laboratory tests and metabolic panel. Plasma glucose levels were measured by the YSI 2300 STAT plus (YSI INC., Yellow Sprigs, OH) in the CSIU. Serum insulin was measured at the Immunochemical Core Laboratory (ICL) at the Mayo Clinic in Rochester. The d2-glucose enrichment data from the euglycemic hyperinsulinemic clamp were measured at the Center for Clinical and Translational Science (CCaTS) Metabolomics Core at the Mayo Clinic in Rochester.

### Muscle biopsy processing

Homogenization of the muscle biopsy (25 mg) was performed in 1X PBS with the Bullet Blender (Integrated Scientific Solutions, San Diego, CA). DNA was isolated using QIAamp DNA mini kit, as per the manufacturer’s instructions (Qiagen, Valencia, CA). DNA quantity and quality was assessed using agarose gel electrophoresis and spectrophotometer A260/A280 values were determined using the NanoVue (GE Healthcare, UK).

### Reduced representation bisulfite sequencing (RRBS) next generation sequencing

RRBS was performed on DNA at the Mayo Clinic Genotyping Shared Resource facility as previously described [38, 39]. Sequencing data were analyzed using a streamlined analysis and annotation pipeline for reduced representation bisulfite sequencing, SAAP-RRBS [39]. The methylation dataset supporting the conclusions of this article are available in the Gene Expression Omnibus repository, GSE164305 (http://www.ncbi.nlm.nih.gov/geo/).

### Differentially methylated cytosines (DMC) analysis

To determine differences in methylation sites between groups, the aligned (Hg19) data were imported into the free open source R package, MethylSig [40]. A minimum of five reads and the recovery of the site in all participants from each group were required for the inclusion of a cytosine in subsequent analyses. The mean methylation differences (%) were determined as previously described, with a Benjamini–Hochberg multiple testing correction applied to the data. Regional annotations for each DMC were imported from the University of California, Santa Cruz (UCSC) Genome Browser’s RefSeq Genes and CpG Island tracks. Priority was given to annotating the site as a promoter or untranslated region if available in another transcript of the gene or in a different gene. The Database for Annotation, Visualization and Integrated Discovery (DAVID) pathway analysis was performed on the DMC data [41, 42].

### ITGB3 quantitative RT-PCR

Skeletal muscle gene expression for *ITGB3* was detected using quantitative real-time PCR on the ABI PRISM 7900HT sequence detection system (Life Technologies, Carlsbad, CA). TaqMan Universal Fast PCR master mix reagents and the Assay-On-Demand gene expression primer pair and probes (Life Technologies, Carlsbad, CA) were added to 50 ng cDNA, which was synthesized using the ABI High Capacity cDNA Reverse Transcription Kit, as per manufacturer’s instructions.

The quantity of *ITGB3* (Hs01001469_m1) in each sample was normalized to 18S (Hs99999901_s1) using the comparative (2-ΔΔCT) method [43].

### ITGB3 luciferase assay

A 1319 bp fragment of the human *ITGB3* promoter (chr17:45,329,911–45,331,229) was cloned into a CpG free luciferase reporter vector (pCpGL-basic). Briefly, the *ITGB3* promoter sequence was identified by searching the gene sequence in the genome web browser ensembl (www.ensembl.org). The transcription start site (TSS) was identified after downloading the gene sequence from the database. We selected the first 1500 bp upstream of the TSS and entered it into the UCSC BLAT (https://genome.ucsc.edu/cgi-bin/hgBlat). We did this to confirm whether the promoter sequence given by ensembl matched the sequence that is annotated in UCSC’s genome browser. The best primer design for the 1500 bp upstream sequence was selected using integrated DNA technologies (IDT) primer design tool (https://www.idtdna.com). The promoter sequence was amplified using the following primers: ITGB3-F-BamHI: ACTAGTGGATCCATACTTGCTGAGGCCAGTGC and ITGB3-R-NcoI: CTTAGTCCATGGAGCCTCACTCACCTCCTACG. The restriction enzymes BamHI and NcoI were used to digest the PCR product, along with the CpG free reporter vector (pCpGL-Basic). The construction of the plasmid was completed by ligating the digestion products using T4 DNA ligase. Propagation of the constructed plasmid was accomplished through transformation of chemically competent GT115 E. coli cells. The sequence of the constructs was validated via DNA sequencing.

The *ITGB3* construct was either mock methylated or methylated using 1600 μM S-adenosylmethionine (SAM) and two different DNA methyltransferases, SssI and HhaI (New England Biolabs, Frankfurt, Germany). Mouse muscle cell lines C2C12 were cultured in DMEM, supplemented with 10% serum and 1% of an antibiotic/antimycotic mixture. Cells were co-transfected with 100 ng of pCpGL-basic with the *ITGB3* promoter insert or without (control) and 2 ng of pRL renilla luciferase control reporter vector using the Lipofectamine 3000 transfection reagent (Invitrogen, Carlsbad, CA). Firefly luciferase activity was measured and normalized against the renilla luciferase activity using the Dual Luciferase Reporter Assay System (Promega, Madison, WI).

### ITGB3 predicted transcription factor binding analysis

Transcription factor binding sites analysis was performed using PROMO version 3.0 [44, 45]. The *ITGB3* sequence on chromosome 17: 45,330,979–45,330,999 encompasses the significant DMC 45,330,989, and that region was assessed for transcription factor binding sites. The sequence was analyzed with a 5% maximum matrix dissimilarity rate using TRANSFAC version 8.3 database.

### Statistical analysis

Data were presented as a mean ± standard error of the mean (SEM). Statistical comparisons of the pre- *versus* post-surgery characteristic data were performed using a paired Student’s *t*-test. Statistical comparisons of the pre-surgery *versus* lean and post-surgery *versus* lean characteristic data was performed using an unpaired Student’s *t*-test. Global methylation data were presented as the means ± SEM. Significance was assessed using a nonparametric Wilcoxon signed-rank test for the paired pre- *versus* post-surgery global methylation analysis. For the unpaired lean *versus* pre-surgery and the unpaired lean *versus* post-surgery global methylation analysis, we performed the nonparametric Mann–Whitney *U* test. MethylSig was used for determining differential methylated cytosines (DMCs) in the reduced representation bisulfite sequencing (RRBS) data. MethylSig uses a beta binomial model to test for significant differences between groups of samples. A comparison of the DNA methylation between the groups at each site was based on a likelihood ratio test (nominal *P* Value), and a Benjamini–Hochberg (*q* < 0.05) multiple testing correction was applied in order to reduce false positives. For the quantitative RT-PCR analyses, the quantity of *ITGB3* was normalized to 18S using the comparative (2-ΔΔCT) method [43] and significance was assessed using a paired Student’s *t*-test. The luciferase data as measured by the luciferase/renilla ratio had a non-normal distribution; therefore, significance across the three groups was determined using the nonparametric Kruskal–Wallis test.

## Supplementary Information

**Additional file 1.** Gene ontology analysis on the genes with significantly decreased DMC in the post-surgery data versus pre-surgery.

**Additional file 2.** Gene ontology analysis on the genes with significantly increased DMC in the post-surgery data versus pre-surgery.

**Additional file 3.** Differentially methylated cytosines (n = 2978) significantly altered in methylation pre-surgery obese versus lean (Benjamini Hochberg *q* < 0.05).

**Additional file 4.** KEGG pathway analysis on the genes with significantly decreased DMC in the pre-surgery obese versus lean.

**Additional file 5.** KEGG pathway analysis on the genes with significantly increased DMC in the pre-surgery obese versus lean.

**Additional file 6.** Differentially methylated cytosines (n = 2885) significantly altered in methylation post-surgery obese versus lean (Benjamini Hochberg *q* < 0.05).

**Additional file 7.** KEGG pathway analysis on the genes with significantly decreased DMC in the post-surgery obese versus lean.

**Additional file 8.** KEGG pathway analysis on the genes with significantly increased DMC in the post-surgery obese versus lean.

## Data Availability

The methylation dataset supporting the conclusions of this article are available in the Gene Expression Omnibus repository, GSE164305 (http://www.ncbi.nlm.nih.gov/geo/).

## Abbreviations

DMC: Differentially methylated cytosine
RYGB: Roux-en-Y gastric bypass
RRBS: Reduced representation bisulfite sequencing
ITGB3: Integrin beta 3
BMI: Body mass index
LDL: Low-density lipoprotein
FPG: Fasting plasma glucose
FSI: Fasting serum insulin
HDL: High-density lipoprotein
HbA1c: Hemoglobin A1c
M-value: Insulin-stimulated glucose disposal
