# Supplementary material for: Weight loss after Roux-En-Y gastric bypass surgery reveals skeletal muscle DNA methylation changes
Source: Clin Epigenetics. 2021 May 1;13:100. doi: 10.1186/s13148-021-01086-6 (PMC8088644; doi:10.1186/s13148-021-01086-6)
Supplement: Supplementary file 1 — Additional file 1. Gene ontology analysis on the genes with significantly decreased DMC in the post-surgery data versus pre-surgery. [file 13148_2021_1086_MOESM1_ESM.docx]

**Additional File 1.** Gene ontology analysis on the genes with significantly decreased DMC in the post-surgery data *versus* pre-surgery

| **Category** | **Term** | **P Value*** | **Genes** | **Fold Enrichment** |
| --- | --- | --- | --- | --- |
| Molecular Function | GO:0003677 ~DNA binding | 0.0027 | TNFRSF6B, ZNF275, NDN, PRR12, TBX1, POLRMT, ISL2, HOXC12, HOXA3, HOXA4, ZNF317, SIM1, PITX2 | 2.41 |
| Biological Processes | GO:0051252 ~regulation of RNA metabolic process | 0.0036 | ZNF275, ISL2, HOXC12, HOXA3, HOXA4, NDN, ZNF317, CASK, TBX1, PABPC1, SIM1, PITX2 | 2.56 |
| Biological Processes | GO:0007389 ~pattern specification process | 0.0043 | SEMA5A, HOXA3, HOXA4, TBX1, PITX2 | 7.24 |
| Biological Processes | GO:0048562 ~embryonic organ morphogenesis | 0.0044 | HOXA3, HOXA4, ALDH1A3, TBX1 | 11.62 |
| Biological Processes | GO:0006928 ~cell motion | 0.0063 | SEMA5A, ISL2, NDN, LYST, MAPK8IP3, TBX1 | 4.88 |
| Biological Processes | GO:0048704 ~embryonic skeletal system morphogenesis | 0.0090 | HOXA3, HOXA4, TBX1 | 20.34 |
| Biological Processes | GO:0048568 ~embryonic organ development | 0.0091 | HOXA3, HOXA4, ALDH1A3, TBX1 | 8.99 |
| Biological Processes | GO:0006355 ~regulation of transcription, DNA-dependent | 0.0097 | ZNF275, ISL2, HOXC12, HOXA3, HOXA4, NDN, ZNF317, CASK, TBX1, SIM1, PITX2 | 2.40 |
| Biological Processes | GO:0048666 ~neuron development | 0.0099 | SEMA5A, ISL2, FSCN2, NDN, MAPK8IP3 | 5.70 |
| Molecular Function | GO:0043565 ~sequence-specific DNA binding | 0.0103 | ISL2, HOXC12, HOXA3, HOXA4, TBX1, PITX2 | 4.28 |
| Biological Processes | GO:0007409 ~axonogenesis | 0.0124 | SEMA5A, ISL2, NDN, MAPK8IP3 | 8.01 |
| Biological Processes | GO:0035270 ~endocrine system development | 0.0129 | HOXA3, ALDH1A3, TBX1 | 16.80 |
| Biological Processes | GO:0060017 ~parathyroid gland development | 0.0150 | HOXA3, TBX1 | 128.84 |
| Biological Processes | GO:0048667 ~cell morphogenesis involved in neuron differentiation | 0.0153 | SEMA5A, ISL2, NDN, MAPK8IP3 | 7.40 |
| Biological Processes | GO:0048706 ~embryonic skeletal system development | 0.0159 | HOXA3, HOXA4, TBX1 | 15.06 |
| Biological Processes | GO:0048812 ~neuron projection morphogenesis | 0.0161 | SEMA5A, ISL2, NDN, MAPK8IP3 | 7.26 |
| Molecular Function | GO:0003700 ~transcription factor activity | 0.0188 | ISL2, HOXC12, HOXA3, HOXA4, TBX1, SIM1, PITX2 | 3.11 |
| Biological Processes | GO:0000904 ~cell morphogenesis involved in differentiation | 0.0230 | SEMA5A, ISL2, NDN, MAPK8IP3 | 6.34 |
| Biological Processes | GO:0048858 ~cell projection morphogenesis | 0.0232 | SEMA5A, ISL2, NDN, MAPK8IP3 | 6.31 |
| Biological Processes | GO:0030182 ~neuron differentiation | 0.0233 | SEMA5A, ISL2, FSCN2, NDN, MAPK8IP3 | 4.41 |
| Biological Processes | GO:0031175 ~neuron projection development | 0.0260 | SEMA5A, ISL2, NDN, MAPK8IP3 | 6.04 |
| Biological Processes | GO:0032990 ~cell part morphogenesis | 0.0260 | SEMA5A, ISL2, NDN, MAPK8IP3 | 6.04 |
| Biological Processes | GO:0007411 ~axon guidance | 0.0295 | SEMA5A, ISL2, MAPK8IP3 | 10.84 |
| Biological Processes | GO:0030878 ~thyroid gland development | 0.0298 | HOXA3, TBX1 | 64.42 |
| Biological Processes | GO:0060541 ~respiratory system development | 0.0300 | ALDH1A3, ANO1, MAPK8IP3 | 10.74 |
| Biological Processes | GO:0048705 ~skeletal system morphogenesis | 0.0321 | HOXA3, HOXA4, TBX1 | 10.35 |
| Biological Processes | GO:0048598 ~embryonic morphogenesis | 0.0413 | HOXA3, HOXA4, ALDH1A3, TBX1 | 5.04 |
| Biological Processes | GO:0048732 ~gland development | 0.0450 | HOXA3, ALDH1A3, TBX1 | 8.59 |
| Biological Processes | GO:0009952 ~anterior/posterior pattern formation | 0.0481 | HOXA3, HOXA4, TBX1 | 8.28 |
| Biological Processes | GO:0045449 ~regulation of transcription | 0.0483 | ZNF275, ISL2, HOXC12, HOXA3, HOXA4, NDN, ZNF317, SETD1A, CASK, TBX1, SIM1, PITX2 | 1.78 |
| Biological Processes | GO:0043009 ~chordate embryonic development | 0.0497 | HOXA3, HOXA4, MAPK8IP3, TBX1 | 4.67 |

Gene ontology analysis performed in DAVID (<https://david.ncifcrf.gov/>). Data organized by P value significance. *P value is uncorrected.
