# Supplementary material for: Weight loss after Roux-En-Y gastric bypass surgery reveals skeletal muscle DNA methylation changes
Source: Clin Epigenetics. 2021 May 1;13:100. doi: 10.1186/s13148-021-01086-6 (PMC8088644; doi:10.1186/s13148-021-01086-6)
Supplement: Supplementary file 2 — Additional file 2. Gene ontology analysis on the genes with significantly increased DMC in the post-surgery data versus pre-surgery. [file 13148_2021_1086_MOESM2_ESM.docx]

**Additional File 2.** Gene ontology analysis on the genes with significantly increased DMC in the post-surgery data *versus* pre-surgery

| **Category** | **Term** | **P Value*** | **Genes** | **Fold Enrichment** |
| --- | --- | --- | --- | --- |
| Molecular function | GO:0031072 ~heat shock protein binding | 0.004 | FAF1, DNAJC5G, DNAJC3 | 30.96 |
| Biological Processes | GO:0008219 ~cell death | 0.013 | OBSCN, AR, FAF1, TRAF6, NLRP1 | 4.95 |
| Biological Processes | GO:0016265 ~death | 0.014 | OBSCN, AR, FAF1, TRAF6, NLRP1 | 4.92 |
| Biological Processes | GO:0043065 ~positive regulation of apoptosis | 0.018 | OBSCN, FAF1, TRAF6, NLRP1 | 6.62 |
| Biological Processes | GO:0043068 ~positive regulation of programmed cell death | 0.019 | OBSCN, FAF1, TRAF6, NLRP1 | 6.58 |
| Biological Processes | GO:0010942 ~positive regulation of cell death | 0.019 | OBSCN, FAF1, TRAF6, NLRP1 | 6.55 |
| Biological Processes | GO:0046578 ~regulation of Ras protein signal transduction | 0.031 | OBSCN, EVI5, ADAP1 | 10.17 |
| Biological Processes | GO:0051056 ~regulation of small GTPase mediated signal transduction | 0.043 | OBSCN, EVI5, ADAP1 | 8.48 |
| Biological Processes | GO:0006915 ~apoptosis | 0.043 | OBSCN, FAF1, TRAF6, NLRP1 | 4.73 |
| Molecular function | GO:0005083 ~small GTPase regulator activity | 0.044 | OBSCN, EVI5, ADAP1 | 8.36 |
| Biological Processes | GO:0012501 ~programmed cell death | 0.045 | OBSCN, FAF1, TRAF6, NLRP1 | 4.66 |

Gene ontology analysis performed in DAVID (<https://david.ncifcrf.gov/>). Data organized by P value significance. *P value is uncorrected.
