# Supplementary material for: Weight loss after Roux-En-Y gastric bypass surgery reveals skeletal muscle DNA methylation changes
Source: Clin Epigenetics. 2021 May 1;13:100. doi: 10.1186/s13148-021-01086-6 (PMC8088644; doi:10.1186/s13148-021-01086-6)
Supplement: Supplementary file 4 — Additional file 4. KEGG pathway analysis on the genes with significantly decreased DMC in the pre-surgery obese versus lean. [file 13148_2021_1086_MOESM4_ESM.docx]

**Additional File 4.** KEGG pathway analysis on the genes with significantly decreased DMC in the pre-surgery obese *versus* lean

| **Category** | **P Value*** | **Genes** | **Fold Enrichment** |
| --- | --- | --- | --- |
| hsa04810 ~Regulation of actin cytoskeleton | 0.00009 | ARHGEF4, FGF18, FGFR3, APC2, LIMK2, DIAPH1, MAP2K2, PDGFA, BAIAP2, FGF11, PIP5K1C, MYH9, FGF20, VAV2, PAK6, CDC42, ITGA9, PPP1CA, DOCK1, CSK, PIP4K2A | 2.67 |
| hsa04510 ~Focal adhesion | 0.00092 | DIAPH1, PDGFA, PIP5K1C, VAV2, COL5A1, PAK6, VEGFB, CDC42, ITGA9, IGF1R, PPP1CA, DOCK1, CCND3, LAMA5, BCL2, COL6A2, RAPGEF1, PARVB | 2.45 |
| hsa00380 ~Tryptophan metabolism | 0.00293 | AADAT, CYP1A1, HAAO, IL4I1, WARS2, OGDH, INMT | 4.78 |
| hsa04270 ~Vascular smooth muscle contraction | 0.00720 | KCNMA1, PPP1CA, ADORA2A, MAP2K2, GNA11, NPR1, ADRA1A, GNAS, PLA2G2C, CACNA1F, PLA2G2F | 2.69 |
| hsa05200 ~Pathways in cancer | 0.00753 | FGF18, FGFR3, APC2, EPAS1, MAP2K2, PDGFA, RXRA, FGF11, EGLN2, FGF20, GLI3, DAPK3, VEGFB, CDC42, IGF1R, WNT3, LAMA5, BCL2, PAX8, RARB, NOS2, TRAF4 | 1.83 |
| hsa04910 ~Insulin signaling pathway | 0.00965 | PRKAG3, PPP1CA, IRS2, EXOC7, SOCS3, MAP2K2, PRKAR1B, PRKAG2, TSC2, FASN, RAPGEF1, RPTOR | 2.43 |
| hsa04360 ~Axon guidance | 0.01842 | PAK6, ABLIM2, CDC42, SEMA5B, PLXNC1, LIMK2, PLXNA1, UNC5D, CXCL12, SLIT2, NFATC1 | 2.33 |
| hsa04920 ~Adipocytokine signaling pathway | 0.03401 | PRKAG3, PPARA, IRS2, SOCS3, STK11, RXRA, PRKAG2 | 2.86 |
| hsa04144 ~Endocytosis | 0.03504 | FGFR3, PIP5K1C, ASAP3, LDLRAP1, RAB11FIP4, IGF1R, CDC42, AP2A2, RAB11FIP3, ACAP3, ARRB1, AGAP1, EPN2 | 1.93 |
| hsa04730 ~Long-term depression | 0.03851 | IGF1R, MAP2K2, GNA11, RYR1, GNAS, PLA2G2C, PLA2G2F | 2.77 |
| hsa05211 ~Renal cell carcinoma | 0.04090 | PAK6, VEGFB, CDC42, EPAS1, MAP2K2, EGLN2, RAPGEF1 | 2.73 |

KEGG analysis performed in DAVID (<https://david.ncifcrf.gov/>). Data organized by P value significance. *P value is uncorrected.
