# Supplementary material for: Weight loss after Roux-En-Y gastric bypass surgery reveals skeletal muscle DNA methylation changes
Source: Clin Epigenetics. 2021 May 1;13:100. doi: 10.1186/s13148-021-01086-6 (PMC8088644; doi:10.1186/s13148-021-01086-6)
Supplement: Supplementary file 5 — Additional file 5. KEGG pathway analysis on the genes with significantly increased DMC in the pre-surgery obese versus lean. [file 13148_2021_1086_MOESM5_ESM.docx]

**Additional File 5.** KEGG pathway analysis on the genes with significantly increased DMC in the pre-surgery obese group *versus* lean

| **Category** | **P Value*** | **Genes** | **Fold Enrichment** |
| --- | --- | --- | --- |
| hsa04720 ~Long-term potentiation | 0.015 | PRKCA, PPP1CA, CALML3, RPS6KA2, GRIN2C, RAP1A, CALML6, PRKCB | 3.05 |
| hsa04070 ~Phosphatidylinositol signaling system | 0.022 | PRKCA, INPP5K, CALML3, PLCG2, PIP5K1C, CALML6, INPP5A, PRKCB | 2.80 |
| hsa04062 ~Chemokine signaling pathway | 0.027 | PARD3, ADCY2, BCAR1, ADCY5, NFKBIB, PREX1, CCNL2, PRKCB, ELMO1, CXCL13, GNB5, RAP1A, WASL, GNG7 | 1.94 |
| hsa04916 ~Melanogenesis | 0.035 | PRKCA, WNT7B, ADCY2, CALML3, ADCY5, CALML6, WNT9A, TCF7L2, PRKCB | 2.36 |
| hsa04020 ~Calcium signaling pathway | 0.037 | PRKCA, ATP2B2, ATP2B3, ADCY2, ATP2B4, SLC25A31, PTGER3, CALML3, GRIN2C, PLCG2, CACNA1H, CALML6, PRKCB | 1.92 |
| hsa04510 ~Focal adhesion | 0.044 | PRKCA, LAMA1, PPP1CA, COL4A1, DOCK1, PDGFB, FLT4, BCAR1, GRLF1, RAP1A, PIP5K1C, ITGB3, FLNA, PRKCB | 1.81 |

KEGG analysis performed in DAVID (<https://david.ncifcrf.gov/>). Data organized by P value significance. *P value is uncorrected
