# Supplementary material for: Weight loss after Roux-En-Y gastric bypass surgery reveals skeletal muscle DNA methylation changes
Source: Clin Epigenetics. 2021 May 1;13:100. doi: 10.1186/s13148-021-01086-6 (PMC8088644; doi:10.1186/s13148-021-01086-6)
Supplement: Supplementary file 7 — Additional file 7. KEGG pathway analysis on the genes with significantly decreased DMC in the post-surgery obese versus lean. [file 13148_2021_1086_MOESM7_ESM.docx]

**Additional File 7.** KEGG pathway analysis on the genes with significantly decreased DMC in the post-surgery obese *versus* lean

| **Category** | **P Value*** | **Genes** | **Fold Enrichment** |
| --- | --- | --- | --- |
| hsa04810:Regulation of actin cytoskeleton | 1.11E-04 | MAP2K2, ITGB5, ITGB4, SRC, ARPC1B, LIMK2, PDGFA, BAIAP2, FGD1, INS, VAV2, GNA12, FGF18, ITGA8, ARHGEF4, PAK6, PIP5K1C, EZR, FGFR4, DOCK1, FGF12, FGFR3, ITGA9 | 2.50 |
| hsa05200:Pathways in cancer | 1.93E-04 | LAMA5, EPAS1, LAMA1, TCF7, SLC2A1, PDGFA, IGF1R, GLI2, RXRA, GNG7, GNA12, AKT1, IKBKG, BID, RALGDS, WNT3, MAP2K2, NOS2, WNT3A, WNT7B, FZD9, VEGFB, FZD8, LAMB1, TRAF2, RUNX1, BCR, BMP2, ADCY9, TRAF4, PAX8, FGF18, FGF12, FGFR3 | 1.98 |
| hsa04510:Focal adhesion | 2.33E-04 | VASP, LAMA5, COL27A1, COL24A1, ITGB5, ITGB4, SRC, LAMA1, RASGRF1, VEGFB, PDGFA, LAMB1, VAV2, IGF1R, PARVG, COL5A1, ITGA8, FLNA, AKT1, PAK6, DOCK1, ITGA9 | 2.44 |
| hsa04512:ECM-receptor interaction | 0.0013 | LAMA5, COL27A1, COL5A1, COL24A1, ITGB5, ITGB4, LAMA1, ITGA8, SDC1, LAMB1, AGRN, ITGA9 | 3.15 |
| hsa04151:PI3K-Akt signaling pathway | 0.0021 | YWHAE, LAMA5, ITGB5, ITGB4, LAMA1, PDGFA, IGF1R, INS, RPTOR, RXRA, CREB3L3, GNG7, CREB3L1, AKT1, IKBKG, MAP2K2, COL27A1, COL24A1, VEGFB, LAMB1, EFNA2, COL5A1, FGF18, ITGA8, FGFR4, FGF12, FGFR3, ITGA9 | 1.85 |
| hsa04931:Insulin resistance | 0.0025 | MLXIP, PTPN1, SLC2A1, NR1H3, PRKAG2, IRS2, ACACB, INS, SOCS3, CREB3L3, CREB3L1, RPS6KA2, AKT1 | 2.75 |
| hsa04390:Hippo signaling pathway | 0.0060 | YWHAE, TEAD4, WNT3A, WNT7B, TCF7, FZD9, FZD8, GDF6, BMP7, STK3, GLI2, BMP2, DLG1, WNT3, LLGL2 | 2.27 |
| hsa05217:Basal cell carcinoma | 0.0085 | BMP2, WNT3A, WNT7B, TCF7, FZD9, FZD8, WNT3, GLI2 | 3.39 |
| hsa04014:Ras signaling pathway | 0.0096 | MAP2K2, PLA2G2C, RASGRF1, VEGFB, PDGFA, KSR2, INS, IGF1R, PLA2G16, EFNA2, GNG7, FGF18, AKT1, PAK6, IKBKG, FGFR4, RALGDS, FGF12, FGFR3 | 1.92 |
| hsa04015:Rap1 signaling pathway | 0.0100 | VASP, MAP2K2, SRC, VEGFB, PDGFA, SIPA1L2, SIPA1L3, RAP1GAP, INS, IGF1R, ADCY9, EFNA2, FGF18, AKT1, FGFR4, RALGDS, FGF12, FGFR3 | 1.96 |
| hsa04920:Adipocytokine signaling pathway | 0.0107 | SOCS3, RXRA, SLC2A1, PRKAG2, AKT1, TRAF2, IRS2, IKBKG, ACACB | 2.94 |
| hsa04916:Melanogenesis | 0.0115 | CAMK2B, ADCY9, MAP2K2, CREB3L3, WNT3A, CREB3L1, WNT7B, TCF7, FZD9, FZD8, WNT3 | 2.51 |
| hsa04974:Protein digestion and absorption | 0.0141 | SLC9A3, COL18A1, COL27A1, COL5A1, COL24A1, COL14A1, ELN, KCNQ1, COL9A3, SLC8A1 | 2.60 |
| hsa04010:MAPK signaling pathway | 0.0142 | MAP2K2, RASGRF1, PDGFA, NFATC1, TRAF2, CACNA2D4, MAPK8IP3, CACNA1H, DUSP6, STK3, RPS6KA2, GNA12, FGF18, FLNA, AKT1, IKBKG, MAPT, FGFR4, FGF12, FGFR3 | 1.81 |
| hsa04550:Signaling pathways regulating pluripotency of stem cells | 0.0188 | MAP2K2, WNT3A, WNT7B, PCGF3, FZD9, FZD8, IGF1R, BMP2, AKT1, FGFR4, FGFR3, NODAL, WNT3 | 2.12 |
| hsa04360:Axon guidance | 0.0224 | SEMA6C, SEMA5B, EFNA2, UNC5A, FES, LIMK2, SEMA3B, LRRC4, PLXNA1, PAK6, PLXNB1, UNC5D | 2.16 |
| hsa04724:Glutamatergic synapse | 0.0266 | GRM4, ADCY9, GNG7, GRIK5, GRIK3, SLC1A2, GRIN2C, SLC1A7, SHANK3, SHANK2, SHANK1 | 2.21 |
| hsa04911:Insulin secretion | 0.0313 | CAMK2B, ADCY9, RAB3A, CREB3L3, CREB3L1, KCNMA1, SLC2A1, KCNN1, INS | 2.42 |
| hsa05222:Small cell lung cancer | 0.0313 | LAMA5, RXRA, TRAF4, NOS2, LAMA1, AKT1, TRAF2, LAMB1, IKBKG | 2.42 |
| hsa04923:Regulation of lipolysis in adipocytes | 0.0341 | PLA2G16, ADCY9, AKT1, IRS2, ADRB1, TSHB, INS | 2.86 |
| hsa05215:Prostate cancer | 0.0374 | MAP2K2, CREB3L3, CREB3L1, TCF7, PDGFA, AKT1, IKBKG, IGF1R, INS | 2.34 |
| hsa04910:Insulin signaling pathway | 0.0381 | RPTOR, PTPN1, SOCS3, MAP2K2, PRKAR1B, PRKAG2, AKT1, IRS2, PHKA1, ACACB, HK1, INS | 1.99 |
| hsa04152:AMPK signaling pathway | 0.0417 | RPTOR, PFKFB3, CREB3L3, STRADA, CREB3L1, PRKAG2, AKT1, IRS2, ACACB, IGF1R, INS | 2.04 |
| hsa04022:cGMP-PKG signaling pathway | 0.0428 | VASP, MAP2K2, NFATC1, IRS2, ADRB1, SLC8A1, INS, ADCY9, CREB3L3, CREB3L1, KCNMA1, GNA12, AKT1 | 1.88 |

KEGG analysis performed in DAVID (<https://david.ncifcrf.gov/>). Data organized by P value significance. *P value is uncorrected.
