# Supplementary material for: Weight loss after Roux-En-Y gastric bypass surgery reveals skeletal muscle DNA methylation changes
Source: Clin Epigenetics. 2021 May 1;13:100. doi: 10.1186/s13148-021-01086-6 (PMC8088644; doi:10.1186/s13148-021-01086-6)
Supplement: Supplementary file 8 — Additional file 8. KEGG pathway analysis on the genes with significantly increased DMC in the post-surgery obese versus lean. [file 13148_2021_1086_MOESM8_ESM.docx]

**Additional File 8.** KEGG pathway analysis on the genes with significantly increased DMC in the post-surgery obese group *versus* lean

| **Category** | **P Value*** | **Genes** | **Fold Enrichment** |
| --- | --- | --- | --- |
| hsa04020:Calcium signaling pathway | 0.00177 | RYR2, PTGER1, PTGER3, CACNA1A, ATP2B3, HTR2C, ADCY2, CACNA1C, CACNA1H, SLC8A2, GRIN2D, ADRB3, ERBB4 | 2.87 |
| hsa04080:Neuroactive ligand-receptor interaction | 0.02200 | GABBR2, PTGER1, GRIK5, PTGER3, HTR1D, HTR2C, GRIK1, RXFP3, GRIN2D, GRM4, ADRB3, PARD3, LEP, NTSR2 | 2.00 |
| hsa04724:Glutamatergic synapse | 0.02441 | GRM4, GRIK5, CACNA1A, GRIK1, ADCY2, CACNA1C, SHANK1, GRIN2D | 2.77 |
| hsa04024:cAMP signaling pathway | 0.02729 | GABBR2, RYR2, PTGER3, HTR1D, ATP2B3, NFATC1, ADCY2, CACNA1C, GLI3, HCN2, GRIN2D | 2.20 |
| hsa04330:Notch signaling pathway | 0.03186 | NCOR2, NOTCH1, CTBP2, RFNG, HES5 | 4.12 |
| hsa00512:Mucin type O-Glycan biosynthesis | 0.04198 | GALNT11, GALNT14, GALNT2, GALNT9 | 5.10 |

KEGG analysis performed in DAVID (<https://david.ncifcrf.gov/>). Data organized by P value significance. *P value is uncorrected
